# DNA Methylation-Based Prediction of Post-operative Atrial Fibrillation

**DOI:** 10.3389/fcvm.2022.837725

**Published:** 2022-05-10

**Authors:** Matthew A. Fischer, Aman Mahajan, Maximilian Cabaj, Todd H. Kimball, Marco Morselli, Elizabeth Soehalim, Douglas J. Chapski, Dennis Montoya, Colin P. Farrell, Jennifer Scovotti, Claudia T. Bueno, Naomi A. Mimila, Richard J. Shemin, David Elashoff, Matteo Pellegrini, Emma Monte, Thomas M. Vondriska

**Affiliations:** ^1^Department of Anesthesiology and Perioperative Medicine, David Geffen School of Medicine at UCLA, Los Angeles, CA, United States; ^2^Department of Anesthesiology and Perioperative Medicine, University of Pittsburgh, Pittsburgh, PA, United States; ^3^Department of Molecular, Cellular and Developmental Biology, University of California, Los Angeles, Los Angeles, CA, United States; ^4^Division of Cardiac Surgery, Department of Surgery, University of California, Los Angeles, Los Angeles, CA, United States; ^5^Department of Medicine, University of California, Los Angeles, Los Angeles, CA, United States; ^6^Department of Biostatistics, University of California, Los Angeles, Los Angeles, CA, United States; ^7^Department of Physiology, University of California, Los Angeles, Los Angeles, CA, United States

**Keywords:** post-operative atrial fibrillation (POAF), epigenomics, DNA methylation, cardiac surgery, precision medicine

## Abstract

**Background:**

Atrial fibrillation (AF) is the most common sustained cardiac arrhythmia and post-operative atrial fibrillation (POAF) is a major healthcare burden, contributing to an increased risk of stroke, kidney failure, heart attack and death. Genetic studies have identified associations with AF, but no molecular diagnostic exists to predict POAF based on pre-operative measurements. Such a tool would be of great value for perioperative planning to improve patient care and reduce healthcare costs. In this pilot study of epigenetic precision medicine in the perioperative period, we carried out bisulfite sequencing to measure DNA methylation status in blood collected from patients prior to cardiac surgery to identify biosignatures of POAF.

**Methods:**

We enrolled 221 patients undergoing cardiac surgery in this prospective observational study. DNA methylation measurements were obtained from blood samples drawn from awake patients prior to surgery. After controlling for clinical and methylation covariates, we analyzed DNA methylation loci in the discovery cohort of 110 patients for association with POAF. We also constructed predictive models for POAF using clinical and DNA methylation data. We subsequently performed targeted analyses of a separate cohort of 101 cardiac surgical patients to measure the methylation status solely of significant methylation loci in the discovery cohort.

**Results:**

A total of 47 patients in the discovery cohort (42.7%) and 43 patients in the validation cohort (42.6%) developed POAF. We identified 12 CpGs that were statistically significant in the discovery cohort after correcting for multiple hypothesis testing. Of these sites, 6 were amenable to targeted bisulfite sequencing and chr16:24640902 was statistically significant in the validation cohort. In addition, the methylation POAF prediction model had an AUC of 0.79 in the validation cohort.

**Conclusions:**

We have identified DNA methylation biomarkers that can predict future occurrence of POAF associated with cardiac surgery. This research demonstrates the use of precision medicine to develop models combining epigenomic and clinical data to predict disease.

## Introduction

Atrial fibrillation (AF), the most common sustained cardiac arrhythmia, is characterized by rapid, irregular atrial depolarizations that lead to an increased risk of clot formation and subsequent stroke (1). Long-term risks of myocardial ischemia, heart failure and dementia are elevated in patients with AF (2–6). Clinical management of AF involves a combination of pharmacological interventions (such as calcium channel blockers) and interventional techniques (such as radiofrequency ablation) to dampen or eliminate the substrates for arrhythmogenic activity (7).

AF is a frequent arrhythmia after cardiac surgery on cardiopulmonary bypass. Approximately 30–40% of cardiac surgeries are complicated by post-operative atrial fibrillation (POAF), which is associated with greater hospital resource utilization, longer ICU and hospital stays and hence greater healthcare costs (8–10). POAF also significantly elevates the risk for more serious long-term cardiovascular complications including myocardial infarction, cardiac arrest, bleeding, renal failure, stroke and death. For coronary artery bypass grafting (CABG) surgery alone, the Society of Thoracic Surgeons database recorded 161,816 procedures in the United States in 2019, plus thousands more procedures in which mitral or aortic valve replacements were performed alone or concomitant with CABG (11).

DNA methylation frequently occurs on cytosines followed by guanine (CpGs) and has been shown to correlate with gene expression in development and disease (12). DNA methylation has been implicated in diseases such as cancer (13), and recent studies have implicated altered global DNA methylation in cardiovascular disease (14–18). Because it has been shown in the Framingham cohort that DNA methylation in peripheral blood samples is associated with AF in the outpatient setting (19), we sought to find CpGs associated with POAF, a clinically distinct manifestation of AF, after cardiac surgery. It would be useful for mechanistic studies to determine the association of these DNA methylation loci with altered gene expression in left atrial tissue; however, it is not possible to obtain left atrial tissue samples from all patients undergoing cardiac surgical procedures without a separate biopsy of the left atrium, an undue risk to patients who otherwise would not have an incision on the left atrium. In this study, we enroll patients undergoing a variety of cardiac surgical procedures (Supplementary Table 1) because these are the patients we care for in the operating room and for which we would like to develop perioperative epigenetic assessment of POAF risk. Thus, we sought to assess the association of DNA methylation in peripheral blood samples with POAF.

While the genetic contribution to AF has been well studied (20), including identification of sequence variation associated with POAF (21), the epigenetic contribution through DNA methylation remains under-explored and represents an appealing target for biomarker discovery based on the following rationale: DNA methylation is relatively stable but not immutable (it can be modified throughout life and thus may serve as a molecular beacon of modifiable risk), and some features of DNA methylation are conserved across tissue types while still exerting cell type specific effects (14, 22, 23). For example, in one study (14) a subset of DNA methylation loci associated with dilated cardiomyopathy in left ventricular myocardial tissue also displayed conserved methylation associated with dilated cardiomyopathy across other tissues, including peripheral blood samples. In the present study, we sought to identify pre-operative epigenomic biomarkers that could be deployed in a precision medicine context to predict a specific and prevalent post-operative complication. We report herein two prediction models combining pre-operative clinical variables and DNA methylation marks that predict POAF in cardiac surgery patients.

## Materials and Methods

### Patient Selection

After institutional review board approval and written informed consent, adult patients scheduled for elective cardiac and aortic surgery on cardiopulmonary bypass were enrolled in this prospective study at the Ronald Reagan Medical Center at the University of California, Los Angeles (UCLA). We chose to enroll patients undergoing a variety of cardiac surgical procedures because this reflects the patient population we care for and for which we would like to implement DNA methylation-based prediction of adverse post-operative outcomes. The cardiac surgical procedures examined in this study are presented in Supplementary Table 1. Patients were enrolled consecutively. A detailed questionnaire was performed, which recorded known clinical risk factors for POAF such as age, proposed surgery and history of paroxysmal AF. We excluded patients with any of the following: AF at the time of surgery, pre- or post-operative complete heart block, emergent surgery, a history of congenital heart disease, organ transplantation, systemic inflammatory diseases, endocarditis or having cardiac surgery without cardiopulmonary bypass. These exclusions reflect: (1) clinical scenarios where pre-operative epigenetic profiling would not be possible due to insufficient time before the surgical procedure; (2) patients where manifestation of AF would not be clinically evident; and (3) disease processes that would significantly affect the peripheral leukocyte composition (e.g., endocarditis).

We enrolled 128 patients in the discovery cohort and reduced representation bisulfite sequencing (RRBS) data was obtained from pre-operative whole blood samples from all of these patients. However, 13 samples were excluded from the discovery cohort analysis based on clinical criteria: 4 patients had permanent AF, 5 patients had perioperative complete heart block, 1 patient died post-operatively, 2 patients had surgery off of cardiopulmonary bypass and 1 patient had endocarditis. Five patients were excluded for poor bisulfite conversion or poor mappability. In the validation cohort, 104 patients were enrolled whose whole blood samples underwent targeted bisulfite sequencing. Three patients were excluded based on clinical criteria: 1 patient had permanent AF, 1 patient had peri-operative complete heart block and 1 patient had surgery off of cardiopulmonary bypass. Several of the clinical exclusions in the discovery and validation cohorts reflect ongoing epigenomic biobanking for analysis of other perioperative complications that are outside the scope of this current study.

### Occurrence of Post-operative Atrial Fibrillation

Patients were followed prospectively to assess for occurrence of POAF. POAF is defined herein as any occurrence of AF of at least 30 s duration after completion of surgery until hospital discharge. We defined POAF as a binary event because we would like to identify methylation loci associated with the development of POAF rather than those related to sustained AF of longer duration. Patients in the cardiac intensive care unit had continuous electrocardiogram monitoring with both electronic and clinical diagnosis of AF. In addition, patients transferred out of the ICU are monitored via telemetry until discharge from the hospital. To ensure data validity, the occurrence of POAF was cross-checked with the electronic medical record (EMR) and the Society of Thoracic Surgeons (STS) database, which contains outcome data including occurrence of POAF recorded by the surgical and medical teams prior to discharge.

### DNA Methylation Data

**Figure 1:**
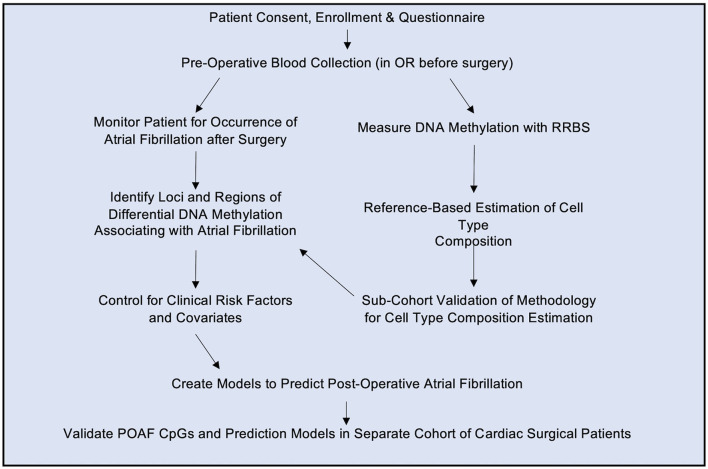
Study Design. Patients giving informed consent were enrolled in the study and a blood sample was drawn in the operating room (OR) prior to surgery. Genomic DNA was isolated from blood and subjected to reduced representation bisulfite sequencing (RRBS). The resulting methylation status was determined as described in the text. Patients were monitored for post-operative atrial fibrillation and differential DNA methylation loci were used to build a model to predict post-operative atrial fibrillation based on pre-operative blood samples. The statistically significant CpGs and predictive model were then analyzed in a separate validation cohort of cardiac surgical patients.

Prior to the start of surgery, 6 mL whole blood samples were drawn from an arterial line in awake patients before induction of anesthesia. Blood samples were collected in a Becton Dickinson pink stoppered EDTA containing tube (Franklin Lakes, NJ), which was stored on ice and processed within 1–3 h. An overall schematic of the experimental workflow is shown in Figure 1. Genomic DNA was isolated (PureLink Genomic DNA Kit, K1820-01) from whole blood (separately we biobanked buffy coat containing leukocytes and platelets at −80°C), subjected to enzymatic digestion with MspI (NEB, R0106S) and bisulfite converted (EpiTect Fast. DNA Bisulfite Kit, Qiagen, 59824). RRBS library preparation was adapted from previous studies (22, 24) using 200 ng of genomic DNA from each sample. In the discovery cohort, libraries were prepared using Illumina TruSeq kit, size-selected for 330 bp with AMPure XP beads and subjected to 100 bp, single-end sequencing on Illumina HiSeq instruments. BS-Seeker2 (25) was used to align the reads to hg38, allowing for up to 10 mismatches, and methylation was called for CpGs and then processed using MethylKit in R as described in previous publications (22, 24). The RRBS approach measures CpG methylation, expressed as the ratio of methylated sequencing reads to all reads for that CpG. We excluded samples with poor bisulfite conversion or mappability.

For the validation cohort, the DNA methylation status of 12 statistically significant CpGs from the discovery cohort were measured in our validation cohort using targeted bisulfite sequencing. As such, the validation cohort was collected after the discovery cohort was completed and the CpGs of interest were identified after analysis of the discovery cohort. The significant CpGs in the discovery cohort had to be identified before sequencing the validation cohort so that DNA probes could be developed to specifically enrich for those same CpGs in the validation cohort using targeted bisulfite sequencing (26). Targeted bisulfite sequencing is less expensive and achieves a higher read depth for the CpGs of interest compared to whole genome bisulfite sequencing or RRBS. For these reasons, targeted bisulfite sequencing is a more focused implementation within a precision medicine environment. For each patient in the validation cohort, 200 ng samples of genomic DNA underwent library preparation involving unique adaptor ligation and were subsequently pooled to be individually sequenced together. Single stranded DNA was then hybridized with 120 bp biotinylated DNA probes designed and synthesized by Integrated DNA Technologies (Coralville, IA). The primer and probe sequences used for targeted bisulfite sequencing (both for validation of RRBS-derived CpGs and for Framingham Heart Study significant CpGs) are included as a Supplementary Excel File. Following biotinylated probe pull down with streptavidin coated magnetic beads, the pooled samples underwent bisulfite conversion, PCR amplification, sequencing and alignment to human reference genome hg38 followed by counting methylated and unmethylated CpGs using BSBolt (27).

### Cell Type Deconvolution

To account for the known confounding of cell type composition on DNA methylation analysis (28), we compared a variety of methods for controlling for cell type heterogeneity. Reference-based deconvolution was performed in the discovery cohort using a method described by Orozco et al. (22), which provides cell type composition estimates using DNA methylation sequencing data. In parallel, reference-free deconvolution was performed on whole blood methylation ratio data using RefFreeEWAS (29) and ReFACTor (30) in R. We separately performed parameter optimization for the reference-free methods and determined that both ReFACTor and RefFreeEWAS optimally modeled 4 surrogates for cell type composition in our dataset.

Fluorescence activated cell sorting (FACS) was performed on a subset (n=14) of patients in the discovery cohort for which additional blood samples were available to assess the accuracy of *in silico* cell type estimation. 5 mL of whole blood was drawn from an arterial line prior to induction of anesthesia at the same time as the blood draw for the methylation data. FACS analysis was performed at the UCLA Immunogenetics Clinical Laboratory to determine percent composition of neutrophils, monocytes, B cells, CD4+ T cells, CD8+ T cells, and NK cells.

### Statistical Analyses

For each sample in the discovery and validation cohorts, we removed from analysis CpGs with <10 × coverage. Additionally, CpGs were removed from the cohort if they were located at SNPs with minor allele frequency >1% in humans (dbSNP build 150 common SNPs: http://hgdownload.cse.ucsc.edu/goldenpath/hg38/database/snp150Common.txt.gz) or not present in at least 50% of the cohort after filtering for coverage.

**Figure 2:**
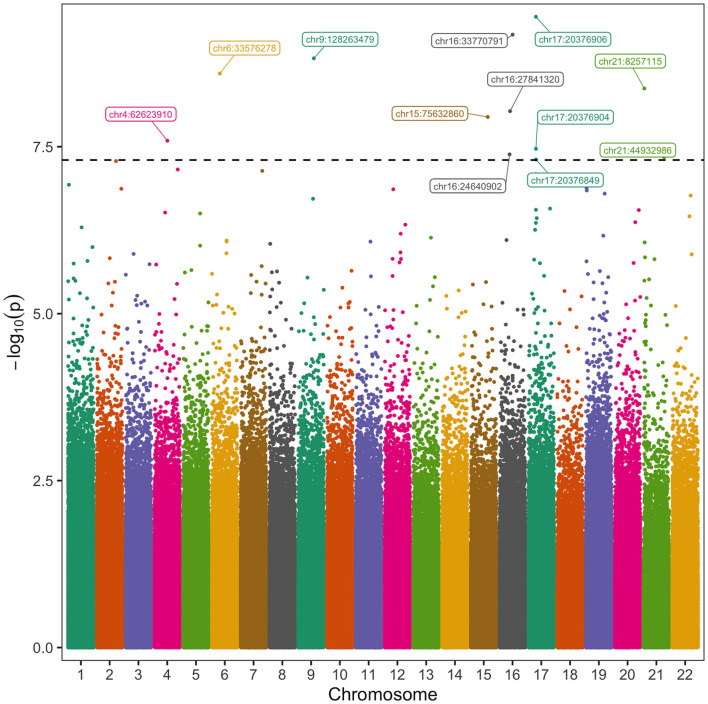
Manhattan Plot of CpGs Associated with Post-operative Atrial Fibrillation. The vertical axis represents the negative log 10 of the *p*-value of each CpG's association with POAF. The horizontal axis represents the chromosome and position of each CpG. Twelve CpGs had significant association with POAF after controlling for known clinical and methylation covariates. The black horizontal dashed line corresponds to a *p*-value of 5 × 10^−8^.

Using the DSS-general package (31) in R version 4.0.2, beta binomial regression was used to regress bisulfite sequencing count data at the filtered CpGs on POAF status. In this regression analysis, we controlled for established clinical covariates (19, 21, 32) (age, sex, history of paroxysmal AF, body mass index, diabetes, hypertension, congestive heart failure, pulmonary vein isolation, MAZE procedure and cardiopulmonary bypass time) and methylation covariates (19) (smoking status, ethnicity and cell type composition estimates) to identify CpGs meeting a predetermined significance threshold (*p*-value < 5 × 10^−8^) for POAF. Patients with more than 2 surgical procedures on cardiopulmonary bypass had a higher incidence of POAF in our cohort so their occurrence was included as a clinical covariate in our analysis. The Manhattan plot in Figure 2 was generated using the ggmanh (33) package in R. The QQ plot was generated using qqman (34) and gglot2 (35) in R. For the validation cohort, we used DSS-general in R to model differential methylation at CpGs highly significant in our discovery cohort and performed regression analysis to determine the association of each CpG with POAF.

The methylation POAF model was constructed and trained using the percent methylation of 3 of the most significant methylation predictors (chr16:24640902, chr16:33770791, and chr21:44932986) and 3 of the most significant clinical predictors (age, history of paroxysmal AF and more than 1 cardiac surgical procedure) in the discovery cohort. The clinical risk factors for this model were selected by stepwise logistic regression in JMP version 16.0.0 (Cary, NC). The methylation model was trained on the discovery cohort using five-fold cross validation repeated 10 times using the caret package in R. The model was then separately tested on the validation cohort to assess model performance in an independent set of samples. An additional methylation POAF prediction model was constructed using chr17:20376906, the CpG within the chr17:20376813-20376906 differentially methylated region (DMR) that was most statistically significant in the discovery cohort, and 4 clinical risk factors (age, history of paroxysmal AF, cardiopulmonary bypass time in minutes and more than 1 cardiac surgical procedure) which were chosen using stepwise logistic regression in JMP. Similarly, this additional methylation model was trained on the discovery cohort using five-fold cross validation repeated 10 times using the caret package in R. Determination of the chr17:20376813-20376906 DMR was made using the callDMR function in DSS-general with default parameters.

In a separate analysis, we performed targeted bisulfite sequencing of published significant CpGs associated with outpatient AF in the Framingham cohort (19). We used the liftOver tool (https://genome.ucsc.edu/cgi-bin/hgLiftOver) to convert hg19 coordinates to hg38 coordinates. We then performed targeted bisulfite sequencing against these loci to determine the methylation status of these CpGs in our validation cohort. We again used DSS-general in R to perform regression analysis to determine the association of each CpG with POAF.

## Results

Of the 110 patients analyzed in the discovery cohort, 47 patients developed POAF for an incidence of 42.7%. There were 101 patients analyzed in the validation cohort, 43 of which experienced POAF for a similar incidence of 42.6%. The incidence in both cohorts is similar to other cardiac surgical populations. The characteristics of the 211 patients analyzed in this study are displayed in Table 1 by cohort. Patients who developed POAF had a longer length of stay with a median difference of an additional 3 days (median of 9 days total vs. 6 days). The difference in length of stay in patients experiencing POAF in all 211 patients was statistically significant (*p*-value < 0.001) as calculated by the Mann-Whitney U test. The surgical procedures for this patient cohort are displayed in Supplementary Table 1. This table shows that patients who had 2 or more surgical procedures (any combination of the following: aortic valve surgery, mitral valve surgery, pulmonic valve surgery, tricuspid valve surgery, CABG surgery, open aortic surgery and myomectomy) on cardiopulmonary bypass had a higher risk of POAF and so this was included as a covariate in our analysis. The racial and ethnic composition of this cohort, another covariate in our analysis, is shown in Supplementary Table 2.

**Table 1:** Characteristics of patient population.

**Characteristic**	**Discovery**		**Validation**	
	**POAF**	**No POAF**	**POAF**	**No POAF**
Total Number of Patients	47 (42.7%)	63 (57.3%)	43 (42.6%)	58 (57.4%)
Age, Mean +/– SD	66.8 +/– 8.8	60.1 +/– 15	68.7 +/– 8.6	58.9 +/– 13.3
Female	15 (31.9%)	22 (34.9%)	8 (18.6%)	21 (36.2%)
History of Paroxysmal Atrial Fibrillation	9 (19.1%)	4 (6.3%)	9 (20.9%)	7 (12.1%)
Diabetes Mellitus	12 (25.5%)	16 (25.4%)	14 (32.6%)	18 (31%)
Hypertension	34 (72.3%)	44 (69.8%)	36 (83.7%)	41 (70.7%)
Congestive Heart Failure	18 (38.3%)	14 (22.2%)	15 (34.9%)	16 (27.6%)
Body Mass Index, Mean +/– SD	29.9 +/– 7.5	27.4 +/– 5.2	28.3 +/– 6.5	28.5 +/– 5.9
Current Smoker	2 (4.3%)	6 (9.5%)	1 (2.3%)	1 (1.7%)
Pulmonary Vein Isolation	0 (0%)	2 (3.2%)	1 (2.3%)	1 (1.7%)
MAZE	6 (12.8%)	2 (3.2%)	4 (9.3%)	4 (6.9%)
Two or More Cardiac Surgical Procedures	24 (51.1%)	20 (31.7%)	22 (51.2%)	15 (25.9%)
Cardiopulmonary Bypass Time, Mean +/– SD	156 +/– 56.9	136.7 +/– 52.7	167.7 +/– 64.7	140.7 +/– 60.9

*The number and prevalence of clinical risk factors in the discovery and validation cohorts are displayed in this table for patients who experience post-operative atrial fibrillation and for those who do not*.

Because DNA methylation at some CpGs is cell-type specific, differences in cell type composition is a known source of confounding which can mistakenly attribute differences in cellular composition as biologic differences related to the disease being studied (28). To account for the heterogeneity of cells in human blood, we compared three methods to control for cell type composition, a reference-based method (22) and two reference-free methods [ReFACTor (30) and RefFreeEWAS (29)]. The reference-based method estimates were the most highly correlated with FACS data so therefore we used reference-based estimates of percent cell type composition as covariates in our analysis of the discovery cohort. Supplementary Table 3 shows the Pearson correlation coefficients between the measured cell type composition and those estimated *in silico* using the reference-based method. The reference-based cell type estimates are statistically significant for neutrophils, monocytes, CD4+ T-cells and NK cells but not B-cells or CD8+ T-cells.

We then analyzed autosomal CpGs in pre-operative blood samples from the discovery cohort for association with POAF while controlling for known clinical and methylation covariates (Figure 2). There were 2,557,388 CpGs with 10 × coverage in at least 50% of patients in our discovery cohort and an average of 2,151,705 of these CpGs per sample. We identified 12 CpGs with strong association with POAF (Table 2). Of these 12 significant CpGs, 7 were within regions of the genome amenable to targeted bisulfite sequencing. CpGs that were not amenable to targeted bisulfite sequencing were within sequences that shared homology with other genomic regions. Six of these CpGs had sequencing data that met sufficient coverage thresholds as defined in the discovery cohort. Of these 6 CpGs, 3 (chr17:20376849, chr17:20376904, and chr17:20376906) are within a differentially methylated region on chromosome 17 and have highly correlated methylation. This DMR, chr17: 20376813-20376906, was identified by the callDMR function in DSS using default parameters and contained a total of 12 CpGs in our discovery cohort dataset. In a separate exercise, we used ReFACTor to analyze our data in an unsupervised manner (Supplementary Table 4). We performed a beta binomial regression controlling for 4 principal components (as determined by the package's parameter optimization tool) and the clinical risk factors as previously described in our initial analysis. Notably, the most important loci (chr16:24640902 and chr17:20376849-20376906) used in our models are still significant after accounting for these principal components.

**Table 2:** CpGs Associated with POAF.

**CpG**	**Methylation**	**Nearest Gene**	**Location**	**Discovery *P*-value**	**Discovery FDR**	**Validation *P*-value**
chr4:62623910	Hypo	ADGRL3-AS1	Upstream	2.57E-08	8.16E-03	
chr6:33576278	Hyper	BAK1	Intron	2.53E-09	1.61E-03	
chr9:128263479	Hyper	GOLGA2	Intron	1.50E-09	1.27E-03	
chr15:75632860	Hyper	IMP3	Downstream	1.13E-08	4.11E-03	
chr16:24640902	Hyper	TNRC6A	Exon	4.11E-08	1.01E-02	0.026
chr16:27841320	Hyper	GSG1L	Intron	9.28E-09	3.93E-03	
chr16:33770791	Hypo	LOC390705	Upstream	6.63E-10	8.42E-04	0.054
chr17:20376849	Hypo	CCDC144CP	Intron	4.91E-08	1.01E-02	0.226
chr17:20376904	Hypo	CCDC144CP	Intron	3.40E-08	9.59E-03	0.690
chr17:20376906	Hypo	CCDC144CP	Intron	3.58E-10	8.42E-04	0.624
chr21:8257115	Hyper	CBS	Intron	4.25E-09	2.16E-03	
chr21:44932986	Hyper	LINC01547	Exon	4.55E-08	1.01E-02	0.078

*Twelve CpGs have p-values <5 × 10^−8^ in the discovery cohort after controlling for clinical and methylation covariates. The CpG methylation status associated with POAF, nearest gene, CpG location relative to the nearest gene and the statistical significance of these loci in the validation cohort are also listed. The methylation status of six CpGs in the validation cohort were unable to be measured using the targeted bisulfite sequencing approach. One CpG, chr16:24640902, was successfully validated in a separate cohort*.

Out of the 6 CpGs significant in the discovery cohort that were able to be sequenced in the validation cohort, one, chr16:24640902, was statistically significant in the validation cohort (*p*-value = 0.026). Chr16:24640902 is within the gene body of TNRC6A, a protein expressed in multiple tissues, including in the left atrium and previously associated with permanent AF (36). The methylation status of chr16:24640902 in patients experiencing POAF and in those that do not is shown in Supplementary Figure 1. Supplementary Table 5 shows the Pearson correlation of CpGs used in the POAF prediction models with cell type estimates calculated *in silico* with the reference-based method for cell type deconvolution. In addition, there is minimal statistical inflation in our analysis with λ = 1.026 (See QQ plot in Supplementary Figure 2).

**Figure 3:**
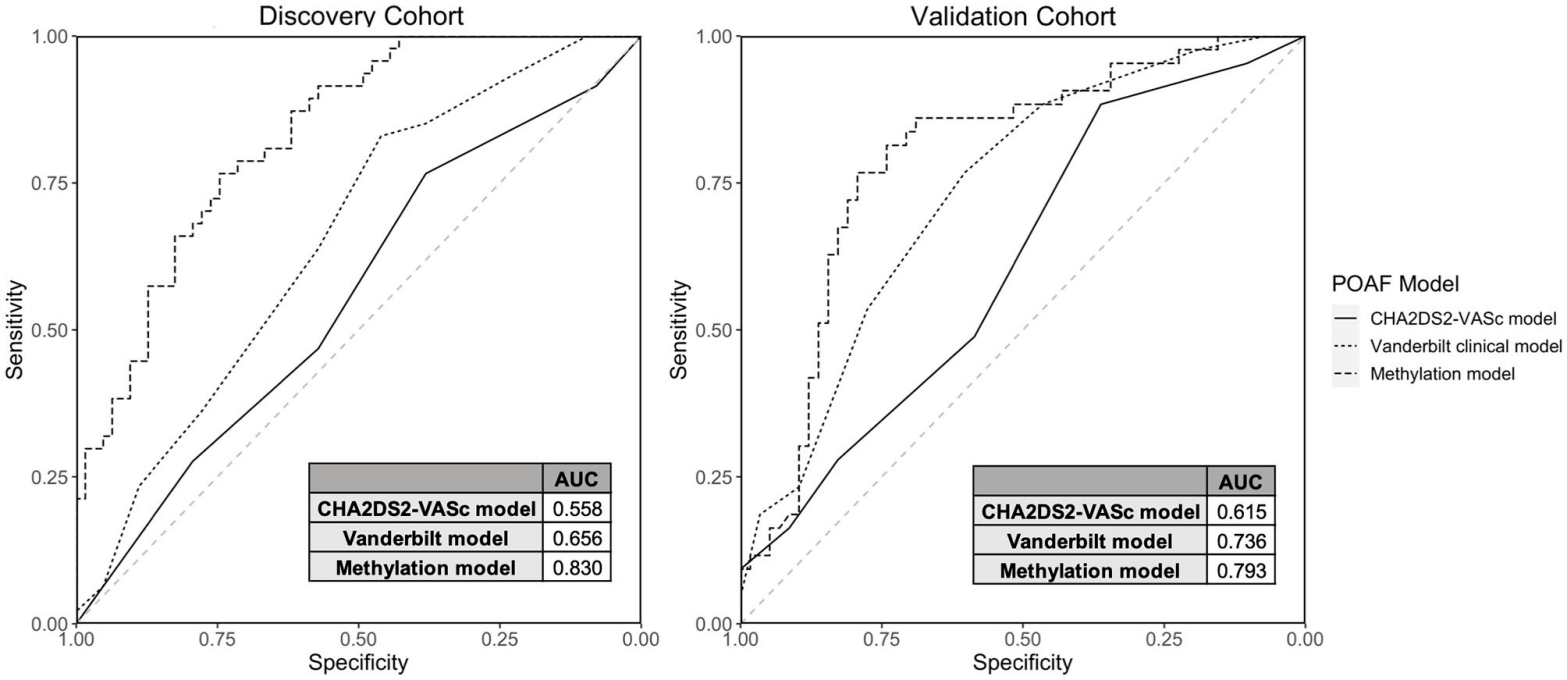
Discovery and Validation Cohort Model Performance. ROC curves and AUCs are shown comparing model performance in the discovery and validation cohorts for the CHA_2_DS_2_-VASc model, the Vanderbilt Cardiac Surgery Registry clinical-only model and the methylation model (3 CpGs + 3 clinical risk factors).

We created two POAF prediction models using clinical risk factors and methylation data. The methylation POAF model (Table 3) was developed using three of the most significant methylation loci and 3 of the most significant clinical risk factors in the discovery cohort. After feature selection in the discovery cohort, the methylation POAF model was trained on discovery cohort data only. The methylation POAF model had an AUC of 0.83 in the discovery cohort and 0.79 in the validation cohort. ROC curves comparing the POAF methylation model performance in the discovery and validation cohorts to published clinical models are shown in Figure 3. The AUC of the methylation loci without clinical risk factors was 0.67 in the validation cohort (data not shown). An additional POAF prediction model (Supplementary Table 6) uses chr17:20376906, the CpG within the chr17: 20376813-20376906 DMR that was most statistically significant in the discovery cohort, and 4 additional clinical risk factors. This model had an AUC of 0.81 in the discovery cohort and 0.67 in the validation cohort.

**Table 3:** Methylation model for post-operative atrial fibrillation.

**Model Parameter**	**Estimate**	**Standard Error**	***P*-value**	**Odd's Ratio**	**95% CI**
**Intercept**	−13.363	3.942			
**Chr16:24640902, per 10% methylation**	10.413	3.415	0.002	2.833	(1.45, 5.53)
**Chr16:33770791, per 10% methylation**	−4.330	1.952	0.027	0.649	(0.44, 0.95)
**Chr21:44932986, per 10% methylation**	6.171	2.906	0.034	1.853	(1.05, 3.28)
**Age, per 10 years**	0.077	0.025	0.002	2.170	(1.33, 3.55)
**History of Paroxysmal Atrial Fibrillation**	2.636	0.932	0.005	13.953	(2.24, 86.77)
**More than 1 Cardiac Surgical Procedure**	0.941	0.494	0.057	2.562	(0.97, 6.74)

*This model uses 3 CpGs and 3 clinical risk factors to predict POAF. This model was constructed using multivariate regression of pre-operative risk factors to predict post-operative atrial fibrillation. Methylation ratios were used instead of the count data used in the primary analysis. Coefficient estimates, odd's ratios, p-values and 95% CIs reflect the significance of each parameter in the format of this model after five-fold cross validation using only the discovery cohort data. The model was then assessed separately on the independent validation cohort*.

We compared the methylation POAF model (Table 3) to two clinical risk factor POAF models: the CHA_2_DS_2_-VASc POAF model (37) and the Vanderbilt Cardiac Surgery Registry clinical-only model (21). CHA_2_DS_2_-VASc, a tool used to predict the risk of embolic stroke in patients with AF, has been shown to predict POAF (37) and has a scoring system that is familiar to most clinicians. The Vanderbilt Cardiac Surgery Registry clinical-only model is a validated prediction model that utilizes seven risk factors for POAF prediction. The CHA_2_DS_2_-VASc model (37) and the Vanderbilt Cardiac Surgery Registry clinical-only model (21) had AUCs of 0.62 and 0.74, respectively, in the validation cohort in our dataset. The methylation POAF model demonstrated a statistically significant improvement in prediction in the validation cohort over the CHA_2_DS_2_-VASc model with *p*-value = 0.00535 by DeLong's test. Compared to the Vanderbilt Cardiac Surgery Registry clinical-only model (21), the methylation POAF model showed a statistically significant improvement in the discovery cohort (*p*-value = 0.00117) but not the validation cohort (*p*-value = 0.264).

In a separate analysis, we assessed the statistical significance of 21 CpGs previously shown to be associated with AF in the Framingham cohort (Supplementary Tables 7, 8) in our validation cohort. In the validation cohort, the most significant Framingham CpG was cg12739419 (*p*-value = 0.033) but it was not statistically significant after accounting for multiple hypothesis testing with the Benjamini-Hochberg procedure (38). The CpG cg12739419, which is known to be associated with the AF SNP rs3807989 and the gene CAV1 in the Framingham cohort, demonstrated hypomethylation associated with POAF, consistent with hypomethylation found at this CpG in the Framingham cohort.

## Discussion

AF is highly prevalent, ranging from 2.7 to 6.1 million affected individuals in the United States and inflicting a cost of ~$6 billion per year on the healthcare system (39). As the most common sustained cardiac arrhythmia, AF is associated with increased risk of stroke, thromboembolism, renal failure, and myocardial infarction. Approximately 40% of people diagnosed with AF or heart failure will develop the other condition (39).

POAF in particular exacerbates morbidity and healthcare costs for patients undergoing what are now routine cardiac surgeries in medical centers around the world (8–10). Previous studies have identified genetic (20) and DNA methylation differences (19, 40) that correlate with AF, but no previous work has provided a molecular measurement that can be made in the blood of presurgical patients that will aid prediction of AF prior to its occurrence. The present study provides such information for the setting of POAF, enabling the development of a simple pre-operative blood test that, when combined with common clinical measurements, can guide the physician in managing cardiac surgery patients according to their relative risk. The prospective design of this study is crucial in that it allows for assessment of epigenetic marks as risk factors for future disease—predominantly in patients without a history of AF—rather than sequelae of disease given the dynamic nature of DNA methylation.

We present a methylation POAF model with 3 clinical factors and 3 CpGs that has an AUC of 0.79 in our validation cohort. We compared this novel prediction model to the CHA_2_DS_2_-VASc POAF model and the Vanderbilt Cardiac Surgery Registry clinical-only model. Though CHA_2_DS_2_-VASc was developed to determine the risk of embolic stroke in patients with AF, it has been shown to predict POAF (37), is simple to implement and has a scoring system that is familiar to most clinicians. The Vanderbilt Cardiac Surgery Registry clinical-only model is a validated prediction model that utilizes seven risk factors for POAF prediction (21). The combined clinical and DNA methylation model had higher AUCs than the CHA_2_DS_2_-VASc model (37) and a leading clinical POAF prediction model (21) which had AUCs of 0.62 and 0.74, respectively, in our validation cohort. The multicenter risk index for atrial fibrillation after cardiac surgery (32) is another important POAF model but was not compared to the methylation model because post-operative risk factors were not a component of the current study.

The methylation POAF model had a statistically significant improvement in prediction over the CHA_2_DS_2_-VASc model in both the discovery and validation cohorts. The methylation POAF model showed statistically improved prediction compared to the Vanderbilt Cardiac Surgery Registry clinical-only model in the discovery cohort only. This demonstrates that DNA methylation can add predictive value to clinical risk factors, though the extent to which this is possible depends on how well the particular clinical risk factors are represented in the patient population in question. The methylation POAF model's performance in the validation cohort shows clearly improved prediction over the CHA_2_DS_2_-VASc model, demonstrating an improvement incorporating DNA methylation biomarkers with clinical predictors. Although the goal of this research was to identify DNA methylation biomarkers that would be applicable to patients who present for a variety of elective cardiac surgical procedures, the diversity of cardiac disease within this cohort such as those with different forms of valvular heart disease may limit the application of these models to cardiac surgical populations which may have a relatively higher proportion of isolated coronary artery bypass grafting procedures compared to combined surgical procedures more typical in the University setting.

In this study, we performed targeted bisulfite sequencing of CpGs associated with AF in the Framingham cohort (19). The finding that the Framingham CpGs were not statistically significant in our validation cohort is consistent with the notion that POAF (not measured in the Framingham study) is a clinically distinct manifestation of AF and has risk factors that are unique compared to AF in the outpatient setting (41). In addition, because we utilized RRBS rather than the Infinium HumanMethylation450 BeadChip Array technology used in the Framingham cohort, the sites associated with POAF in this study were not measured in the Framingham cohort dataset and cannot be analyzed to assess their association with AF in the outpatient setting.

The methodology presented in this study could be implemented in a precision medicine environment where an epigenomic risk metric of POAF and other adverse peri-operative outcomes could be determined from a blood draw with other routine pre-operative labs. In particular, epigenomic risk profiles could be of significant value in perioperative risk stratification and planning perioperative care for each patient. Further research is needed to refine prediction models and associations with additional outcomes of interest. The epigenomic profile of each patient could also help assess perioperative risk and guide clinical decision making for patients at risk for POAF and other adverse outcomes. In addition, it could help guide which drugs or treatments patients may respond better to given their own perioperative risks and epigenomic profile. DNA methylation risk loci could also be targeted directly. In a rat model of spontaneous AF, administration of the DNA methylation inhibitor decitabine reduced atrial tachycardia, demonstrating the possibility of targeting DNA methylation for therapeutic effect (42). It will also be informative to evaluate whether these or other epigenomic marks may serve as biomarkers for AF outside of the context of cardiac surgery. With improvements in sequencing technology, patient samples can be pooled to simultaneously undergo targeted bisulfite sequencing for each individual sample at many loci of interest for reduced cost compared to RRBS and whole genome bisulfite sequencing. Targeted bisulfite sequencing costs $25–50 per sample with results available in ~2 weeks. Targeted bisulfite sequencing would ideally be utilized for patients undergoing surgeries that have a relatively higher risk of adverse outcomes—such as cardiac surgery—and include epigenetic risk factors for multiple adverse outcomes that could be used together for optimal perioperative planning.

Though this is an association study, the prospective study design and utilization of epigenetic risk factors have the potential to identify mechanisms impacting POAF susceptibility with greater granularity than clinical risk factors alone. What are the molecular mechanisms by which the association between DNA methylation and risk of AF is established? DNA methylation in so-called CpG islands is thought to control gene expression by silencing promoters and regulatory regions (43). Gene body methylation, however, is associated with increased transcription (12). The genes nearest to the regions of altered methylation observed in this study (Table 2) provide some hints about a potential molecular mechanism acting in cis to regulate the nearest gene. Chr16:24640902, a CpG statistically significant in both the discovery and validation cohorts, is within the gene body of TNRC6A, a protein expressed in many tissues including the left atrium, associated with miRNA-mediated gene silencing (44) and previously associated with permanent AF (36). The significance of micro-RNAs has emerged in the pathophysiology of AF through electrical and structural remodeling (45). The other CpGs in the POAF prediction model have unknown relation to AF. Overall, these sites may reflect molecular mechanisms in the left atrium that create substrates for POAF, which then become activated by the physiologic stress of cardiopulmonary bypass and surgery. Another mechanism by which altered CpG methylation may control gene expression and organ phenotype is through chromatin structure, recently appreciated to be globally remodeled in the setting of heart failure in human cells and animal models (46, 47). Future studies will be required to resolve the molecular mechanisms definitively—however, this study provides a novel set of biomarkers to be explored clinically in larger cohorts for their ability to aid in the prediction of AF, toward the goal of preventing many of the downstream disease sequelae precipitated by this arrhythmia.

## Data Availability Statement

The datasets presented in this study can be found in online repositories. The names of the repository/repositories and accession number(s) can be found below: GEO; GSE194156.

## Ethics Statement

The studies involving human participants were reviewed and approved by University of California, Los Angeles. The patients/participants provided their written informed consent to participate in this study.

## Author Contributions

MF performed bioinformatic, read mapping/alignment and statistical analyses with contributions from DM, DC, and DE. MC, TK, ES, and EM performed experiments including genomic DNA library preparation. MM, CF, and MP performed bisulfite sequencing. JS, NM, and CB obtained patient data. MF and RS recruited patients and performed clinical assessment. MF, AM, and TV conceived of the study which was directed by MF and TV. MF and TV wrote the manuscript. All authors contributed to the article and approved the submitted version.

## Funding

This project was supported by the UCLA Department of Anesthesiology, the UCLA Cardiovascular Theme and NIH grants UL1TR001881 (UCLA CTSI, PI: Steven Dubinett), HL 105699 (TMV), HL150667 (TMV), HL 115238 (TMV). MF was supported by a Starter Grant from the Society of Cardiovascular Anesthesiologists and a mentored research training grant by the Foundation for Anesthesia Education and Research.

## Conflict of Interest

The authors declare that the research was conducted in the absence of any commercial or financial relationships that could be construed as a potential conflict of interest.

## Publisher's Note

All claims expressed in this article are solely those of the authors and do not necessarily represent those of their affiliated organizations, or those of the publisher, the editors and the reviewers. Any product that may be evaluated in this article, or claim that may be made by its manufacturer, is not guaranteed or endorsed by the publisher.

## References

[1] Morin DP Bernard ML Madias C Rogers PA Thihalolipavan S Estes NA. The state of the art: atrial fibrillation epidemiology, prevention, and treatment. Mayo Clin Proc. (2016) 91:1778–810. 10.1016/j.mayocp.2016.08.022 27825618

[2] Kalantarian S Stern TA Mansour M Ruskin JN. Cognitive impairment associated with atrial fibrillation: A meta-analysis. Ann Intern Med. (2013) 158:338-46. 10.7326/0003-4819-158-5-201303050-00007 23460057 PMC4465526

[3] Lee HY Yang PS Kim TH Uhm JS Pak HN Lee MH. Atrial fibrillation and the risk of myocardial infarction: A nation-wide propensity-matched study. Sci Rep. (2017) 7:12716. 10.1038/s41598-017-13061-4 28983076 PMC5629219

[4] Owan TE Hodge DO Herges RM Jacobsen SJ Roger VL Redfield MM. Trends in prevalence and outcome of heart failure with preserved ejection fraction. N Engl J Med. (2006) 355:251-9. 10.1056/NEJMoa052256 16855265

[5] Bunch TJ. Atrial fibrillation and dementia. Circulation. (2020) 142:618–20. 10.1161/circulationaha.120.045866 32804567

[6] Verma A Kalman JM Callans DJ. Treatment of patients with atrial fibrillation and heart failure with reduced ejection fraction. Circulation. (2017) 135:1547–63. 10.1161/circulationaha.116.026054 28416525

[7] Xu J Luc JG Phan K. Atrial fibrillation: Review of current treatment strategies. J Thorac Dis. (2016) 8:e886–e900. 10.21037/jtd.2016.09.13 27747025 PMC5059277

[8] Greenberg JW Lancaster TS Schuessler RB Melby SJ. Postoperative atrial fibrillation following cardiac surgery: A persistent complication. Eur J Cardiothorac Surg. (2017) 52:665–72. 10.1093/ejcts/ezx039 28369234

[9] D'Agostino RS Jacobs JP Badhwar V Fernandez FG Paone G Wormuth DW. The society of thoracic surgeons adult cardiac surgery database: 2018 update on outcomes and quality. Ann Thorac Surg. (2018) 105:15–23. 10.1016/j.athoracsur.2017.10.035 29233331

[10] Ha AC Mazer CD Verma S Yanagawa B Verma A. Management of postoperative atrial fibrillation after cardiac surgery. Curr Opin Cardiol. (2016) 31:183–90. 10.1097/HCO.0000000000000264 26836987

[11] Bowdish ME D'Agostino RS Thourani VH Schwann TA Krohn C Desai N. Sts adult cardiac surgery database: 2021 update on outcomes, quality, and research. Ann Thorac Surg. (2021) 111:1770–80. 10.1016/j.athoracsur.2021.03.043 33794156

[12] Greenberg MVC Bourc'his D. The diverse roles of dna methylation in mammalian development and disease. Nat Rev Mol Cell Biol. (2019) 20:590–607. 10.1038/s41580-019-0159-6 31399642

[13] Jones PA Issa JP Baylin S. Targeting the cancer epigenome for therapy. Nat Rev Genet. (2016) 17:630–41. 10.1038/nrg.2016.93 27629931

[14] Meder B Haas J Sedaghat-Hamedani F Kayvanpour E Frese K Lai A. Epigenome-wide association study identifies cardiac gene patterning and a novel class of biomarkers for heart failure. Circulation. (2017) 136:1528–44. 10.1161/circulationaha.117.027355 28838933

[15] Movassagh M Choy MK Knowles DA Cordeddu L Haider S Down T. Distinct epigenomic features in end-stage failing human hearts. Circulation. (2011) 124:2411–22. 10.1161/CIRCULATIONAHA.111.040071 22025602 PMC3634158

[16] Huan T Joehanes R Song C Peng F Guo Y Mendelson M. Genome-wide identification of dna methylation qtls in whole blood highlights pathways for cardiovascular disease. Nat Commun. (2019) 10:4267. 10.1038/s41467-019-12228-z 31537805 PMC6753136

[17] Ma J Rebholz CM Braun KVE Reynolds LM Aslibekyan S Xia R. Whole blood dna methylation signatures of diet are associated with cardiovascular disease risk factors and all-cause mortality. Circ Genom Precis Med. (2020) 13:e002766. 10.1161/circgen.119.002766 32525743 PMC7442697

[18] Agha G Mendelson MM Ward-Caviness CK Joehanes R Huan T Gondalia R. Blood leukocyte dna methylation predicts risk of future myocardial infarction and coronary heart disease. Circulation. (2019) 140:645–57. 10.1161/circulationaha.118.039357 31424985 PMC6812683

[19] Lin H Yin X Xie Z Lunetta KL Lubitz SA Larson MG. Methylome-wide association study of atrial fibrillation in framingham heart study. Sci Rep. (2017) 7:40377. 10.1038/srep40377 28067321 PMC5220313

[20] Fatkin D Santiago CF Huttner IG Lubitz SA Ellinor PT. Genetics of atrial fibrillation: State of the art in 2017. Heart Lung Circ. (2017) 26:894-901. 10.1016/j.hlc.2017.04.008 28601532

[21] Kolek MJ Muehlschlegel JD Bush WS Parvez B Murray KT Stein CM. Genetic and clinical risk prediction model for postoperative atrial fibrillation. Circ Arrhythm Electrophysiol. (2015) 8:25–31. 10.1161/circep.114.002300 25567478 PMC4334678

[22] Orozco LD Farrell C Hale C Rubbi L Rinaldi A Civelek M. Epigenome-wide association in adipose tissue from the metsim cohort. Hum Mole Genet. (2018) 27:2586. 10.1093/hmg/ddy205 29893869 PMC6030960

[23] Orozco LD Morselli M Rubbi L Guo W Go J Shi H. Epigenome-wide association of liver methylation patterns and complex metabolic traits in mice. Cell Metab. (2015) 21(6):905–17. 10.1016/j.cmet.2015.04.025 26039453 PMC4454894

[24] Chen H Orozco L Wang J Rau CD Rubbi L Ren S. DNA methylation indicates susceptibility to isoproterenol-inducd cardiac pathology and is associated with chromatin states. Circ Res. (2016) 118:786–97. 10.1161/CIRCRESAHA.115.305298 26838786 PMC4779427

[25] Guo W Fiziev P Yan W Cokus S Sun X Zhang MQ. Bs-Seeker2: A versatile aligning pipeline for bisulfite sequencing data. BMC Genom. (2013) 14:774. 10.1186/1471-2164-14-774 24206606 PMC3840619

[26] Morselli M Farrell C Rubbi L Fehling HL Henkhaus R Pellegrini M. Targeted bisulfite sequencing for biomarker discovery. Methods. (2020) 187:13-27. 10.1016/j.ymeth.2020.07.006 32755621 PMC7855209

[27] Farrell C Thompson M Tosevska A Oyetunde A Pellegrini M. Bisulfite bolt: A bisulfite sequencing analysis platform. bioRxiv. (2021). 10.1101/2020.10.06.328559 33966074 PMC8106542

[28] Jaffe AE Irizarry RA. Accounting for cellular heterogeneity is critical in epigenome-wide association studies. Genome Biol. (2014) 15:R31. 10.1186/gb-2014-15-2-r31 24495553 PMC4053810

[29] Houseman EA Kile ML Christiani DC Ince TA Kelsey KT Marsit CJ. Reference-free deconvolution of dna methylation data and mediation by cell composition effects. BMC Bioinform. (2016) 17:259. 10.1186/s12859-016-1140-4 27358049 PMC4928286

[30] Rahmani E Zaitlen N Baran Y Eng C Hu D Galanter J. Sparse pca corrects for cell type heterogeneity in epigenome-wide association studies. Nat Methods. (2016) 13:443–5. 10.1038/nmeth.3809 27018579 PMC5548182

[31] Park Y Wu H. Differential Methylation Analysis For Bs-Seq data under general experimental design. Bioinformatics. (2016) 32:1446–53. 10.1093/bioinformatics/btw026 26819470 PMC12157722

[32] Mathew JP Fontes ML Tudor IC Ramsay J Duke P Mazer CD. A multicenter risk index for atrial fibrillation after cardiac surgery. JAMA. (2004) 291:1720–9. 10.1001/jama.291.14.1720 15082699

[33] Lee J. Ggmanh: Visualization Tool for Gwas Result. R package version 0.99.12 ed. (2022).

[34] Turner SD. Qqman: An r package for visualizing gwas results using Q-Q and manhattan plots. bioRxiv. (2014). 10.1101/005165

[35] Wickham H. Ggplot2: Elegant Graphics for Data Analysis. New York, NY: Springer-Verlag (2016).

[36] Doñate Puertas R Jalabert A Meugnier E Euthine V Chevalier P Rome S. Analysis of the microrna signature in left atrium from patients with valvular heart disease reveals their implications in atrial fibrillation. PLoS ONE. (2018) 13:e0196666. 10.1371/journal.pone.0196666 29723239 PMC5933750

[37] Kashani RG Sareh S Genovese B Hershey C Rezentes C Shemin R. Predicting postoperative atrial fibrillation using cha2ds2-vasc scores. J Surg Res. (2015) 198:267-72. 10.1016/j.jss.2015.04.047 26004496

[38] Benjamini Y Hochberg Y. Controlling the false discovery rate: a practical and powerful approach to multiple testing. J Royal Statis Soc Ser B. (1995) 57:289-300.

[39] Benjamin EJ Virani SS Callaway CW Chamberlain AM Chang AR Cheng S. Heart disease and stroke statistics-2018 update: A report from the american heart association. Circulation. (2018) 137:e67–492. 10.1161/CIR.0000000000000558 29386200

[40] Zhao G Zhou J Gao J Liu Y Gu S Zhang X. Genome-wide dna methylation analysis in permanent atrial fibrillation. Mol Med Rep. (2017) 16:5505-14. 10.3892/mmr.2017.7221 28849195

[41] Dobrev D AM Heijman J Guichard J Nattel S. Postoperative atrial fibrillation: Mechanisms, manifestations and management. Nat Rev Cardiol. (2019) 16:417-36. 10.1038/s41569-019-0166-5 30792496

[42] Doñate Puertas R Meugnier E Romestaing C Rey C Morel E Lachuer J. Atrial fibrillation is associated with hypermethylation in human left atrium, and treatment with decitabine reduces atrial tachyarrhythmias in spontaneously hypertensive rats. Transl Res. (2017) 184:57–67.e5. 10.1016/j.trsl.2017.03.004 28427903

[43] Jones PA. Functions of DNA methylation: Islands, start sites, gene bodies and beyond. Nat Rev Genet. (2012) 13:484–92. 10.1038/nrg3230 22641018

[44] Nishi K Nishi A Nagasawa T Ui-Tei K. Human Tnrc6a Is an argonaute-navigator protein for microrna-mediated gene silencing in the nucleus. RNA. (2013) 19:17–35. 10.1261/rna.034769.112 23150874 PMC3527724

[45] Nattel S Harada M. Atrial remodeling and atrial fibrillation: Recent advances and translational perspectives. J Am Coll Cardiol. (2014) 63:2335–45. 10.1016/j.jacc.2014.02.555 24613319

[46] Rosa-Garrido M Chapski DJ Schmitt AD Kimball TH Karbassi E Monte E. High-resolution mapping of chromatin conformation in cardiac myocytes reveals structural remodeling of the epigenome in heart failure. Circulation. (2017) 136:1613–25. 10.1161/CIRCULATIONAHA.117.029430 28802249 PMC5648689

[47] Gilsbach R Schwaderer M Preissl S Grüning BA Kranzhöfer D Schneider P. Distinct epigenetic programs regulate cardiac myocyte development and disease in the human heart *in vivo*. Nat Commun. (2018) 9:391. 10.1038/s41467-017-02762-z 29374152 PMC5786002

