# Supplementary material for: DNA Methylation-Based Prediction of Post-operative Atrial Fibrillation
Source: Front Cardiovasc Med. 2022 May 10;9:837725. doi: 10.3389/fcvm.2022.837725 (PMC9127230; doi:10.3389/fcvm.2022.837725)
Supplement: Supplementary file 2 [file Presentation_1.pptx]

## Slide 1
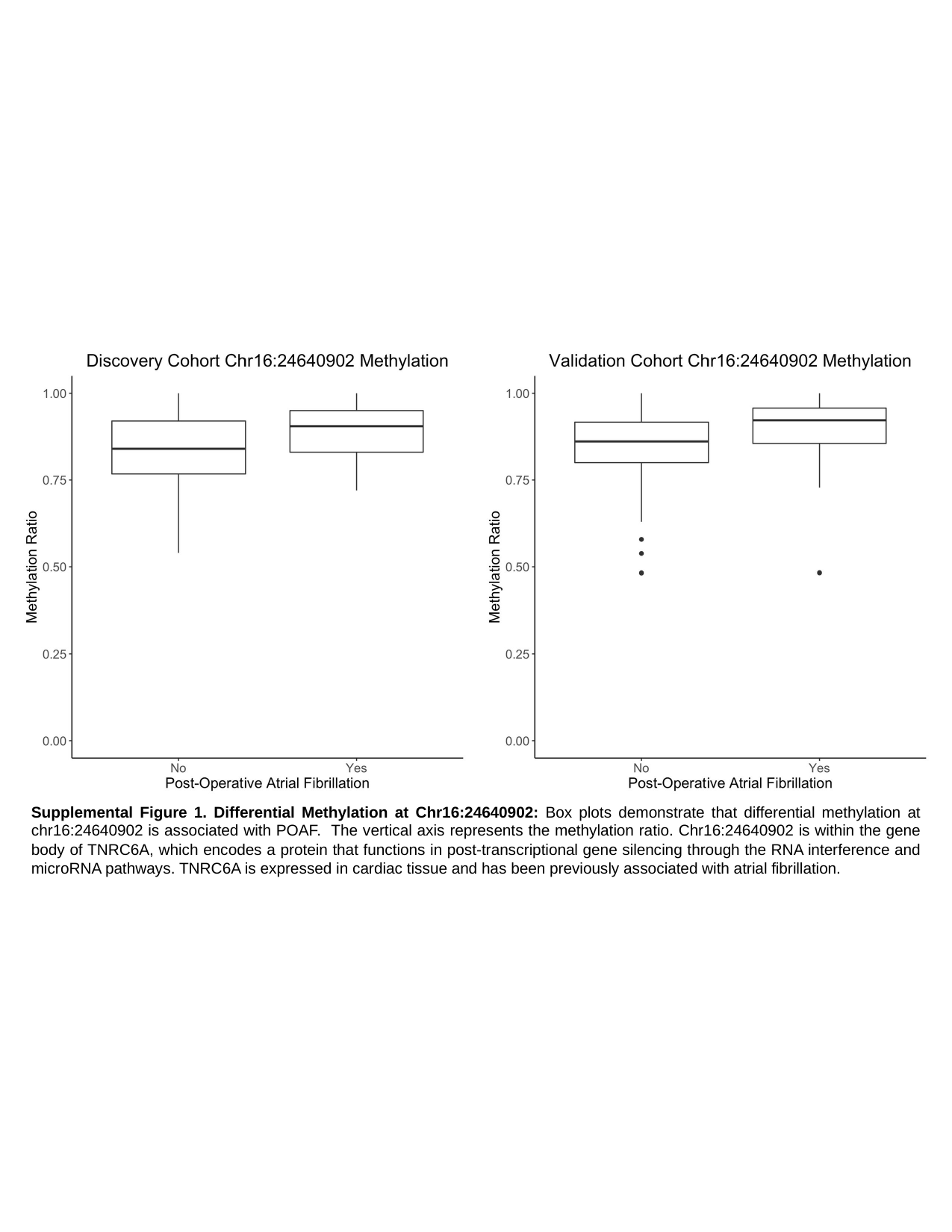

Supplemental Figure 1. Differential Methylation at Chr16:24640902: Box plots demonstrate that differential methylation at chr16:24640902 is associated with POAF. The vertical axis represents the methylation ratio. Chr16:24640902 is within the gene body of TNRC6A, which encodes a protein that functions in post-transcriptional gene silencing through the RNA interference and microRNA pathways. TNRC6A is expressed in cardiac tissue and has been previously associated with atrial fibrillation.

## Slide 2
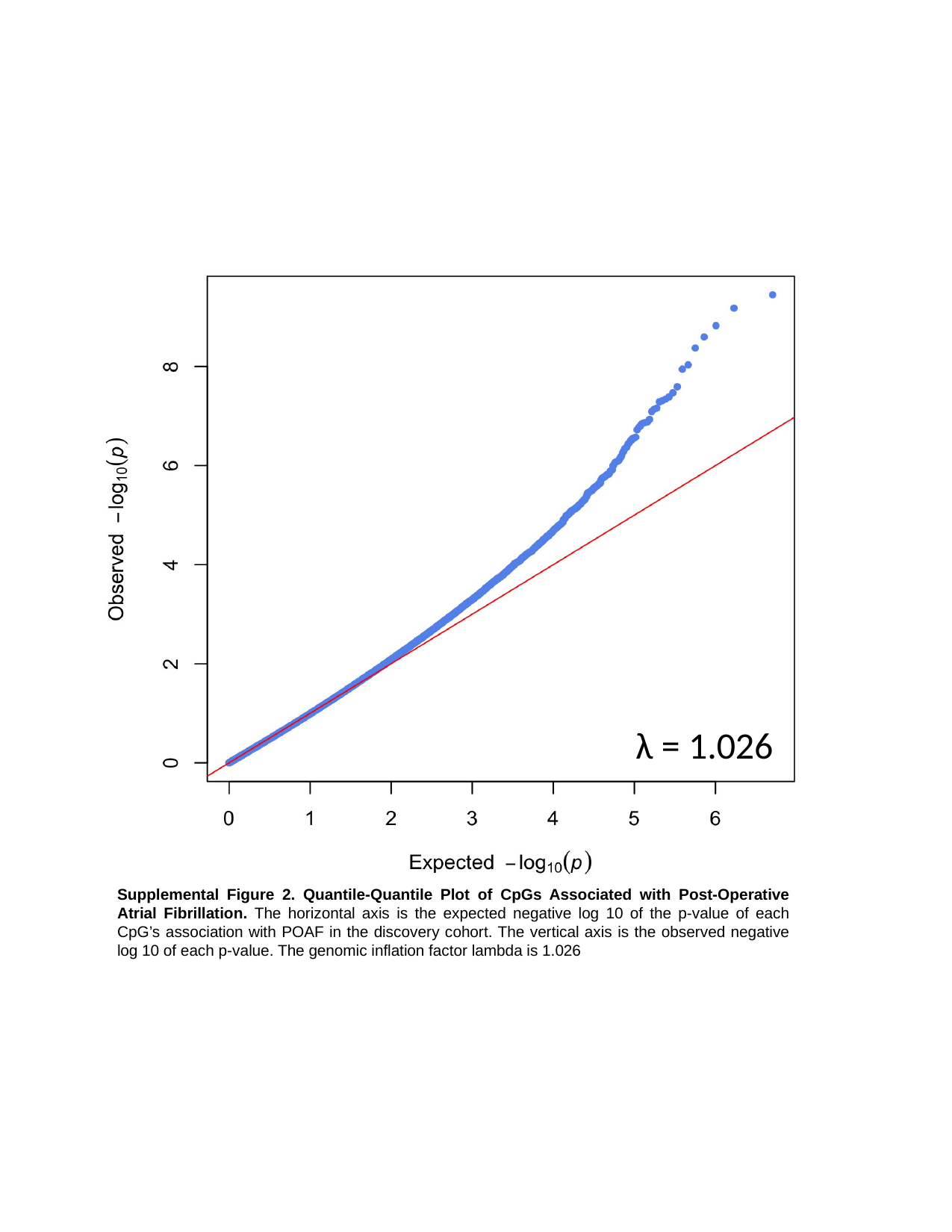

λ = 1.026
Supplemental Figure 2. Quantile-Quantile Plot of CpGs Associated with Post-Operative Atrial Fibrillation. The horizontal axis is the expected negative log 10 of the p-value of each CpG’s association with POAF in the discovery cohort. The vertical axis is the observed negative log 10 of each p-value. The genomic inflation factor lambda is 1.026

## Slide 3
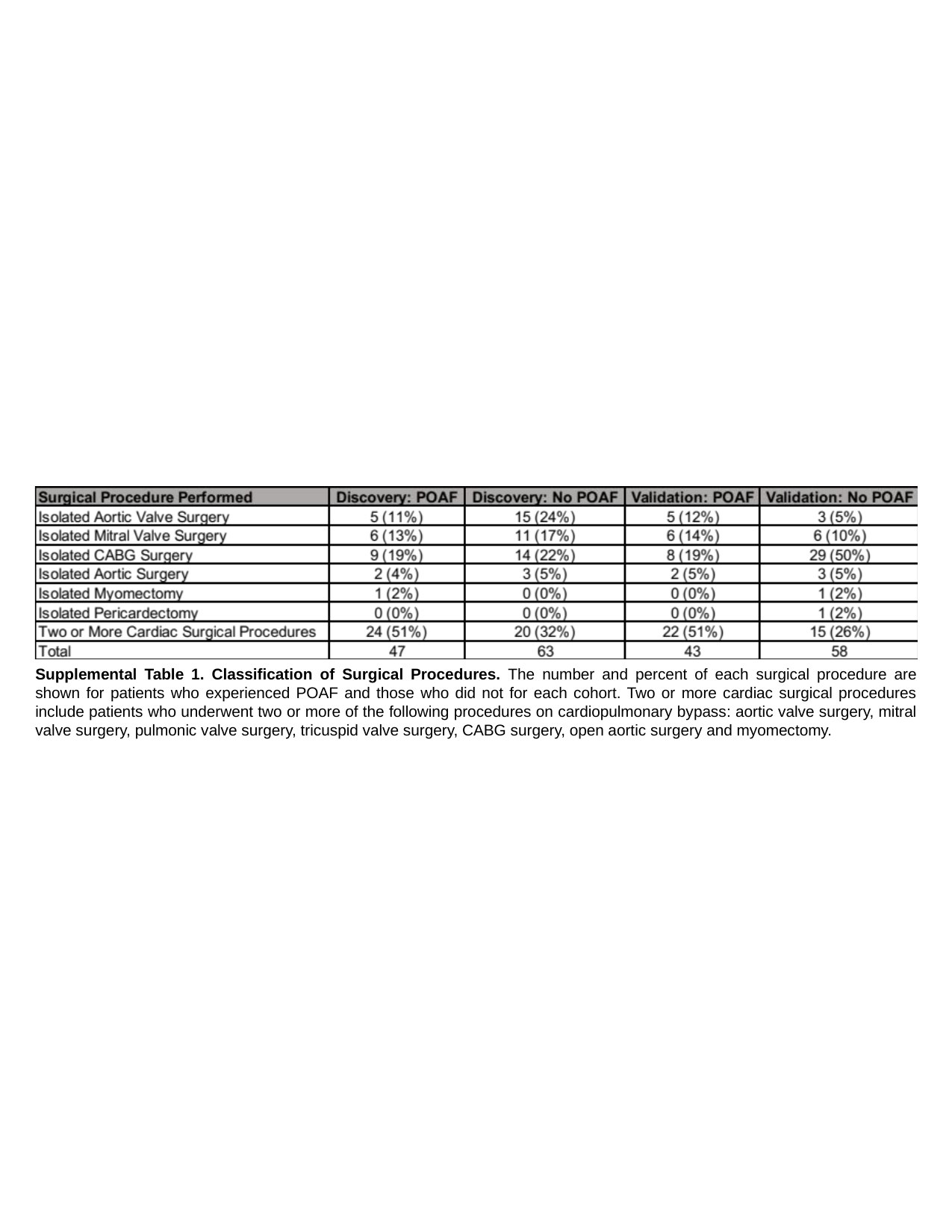

Supplemental Table 1. Classification of Surgical Procedures. The number and percent of each surgical procedure are shown for patients who experienced POAF and those who did not for each cohort. Two or more cardiac surgical procedures include patients who underwent two or more of the following procedures on cardiopulmonary bypass: aortic valve surgery, mitral valve surgery, pulmonic valve surgery, tricuspid valve surgery, CABG surgery, open aortic surgery and myomectomy.

## Slide 4
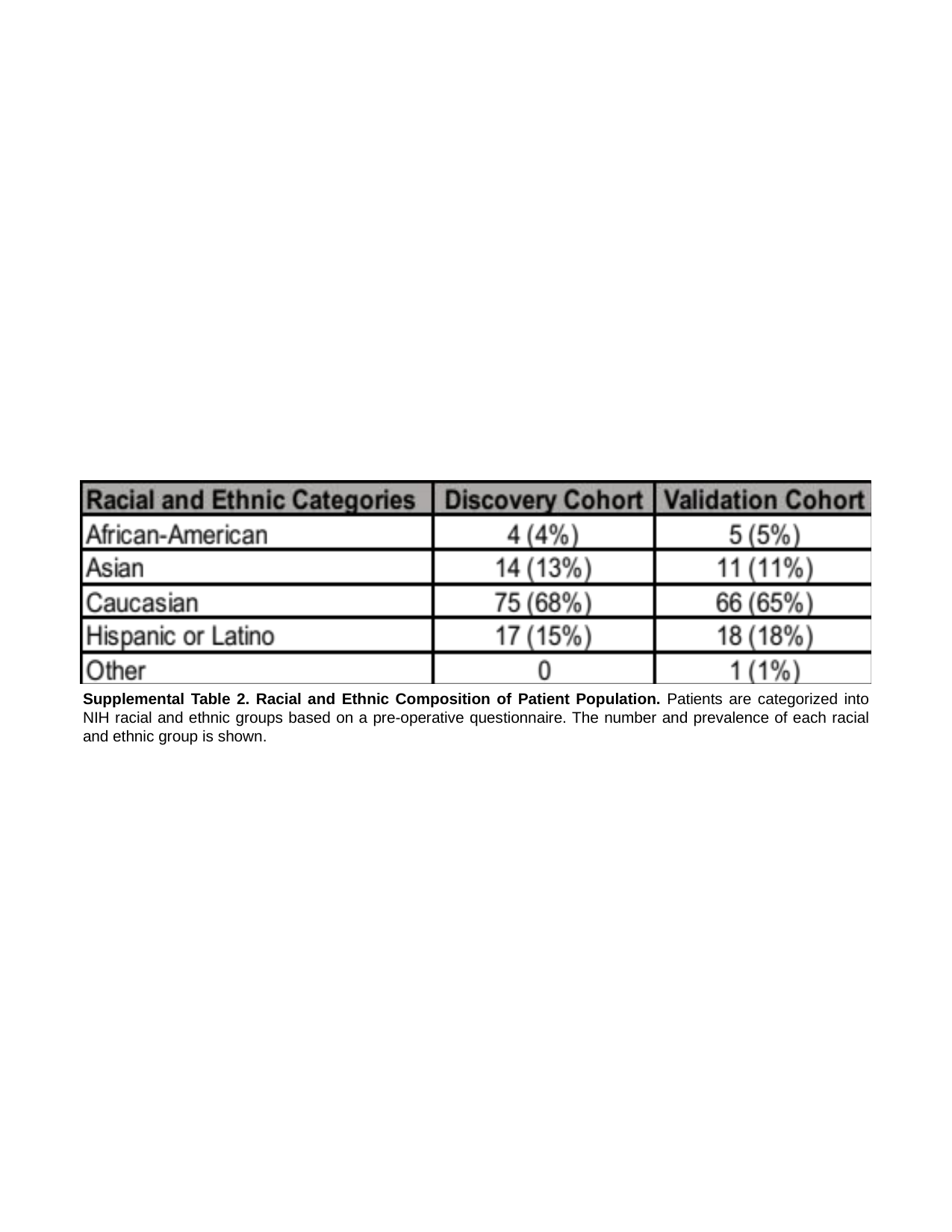

Supplemental Table 2. Racial and Ethnic Composition of Patient Population. Patients are categorized into NIH racial and ethnic groups based on a pre-operative questionnaire. The number and prevalence of each racial and ethnic group is shown.

## Slide 5
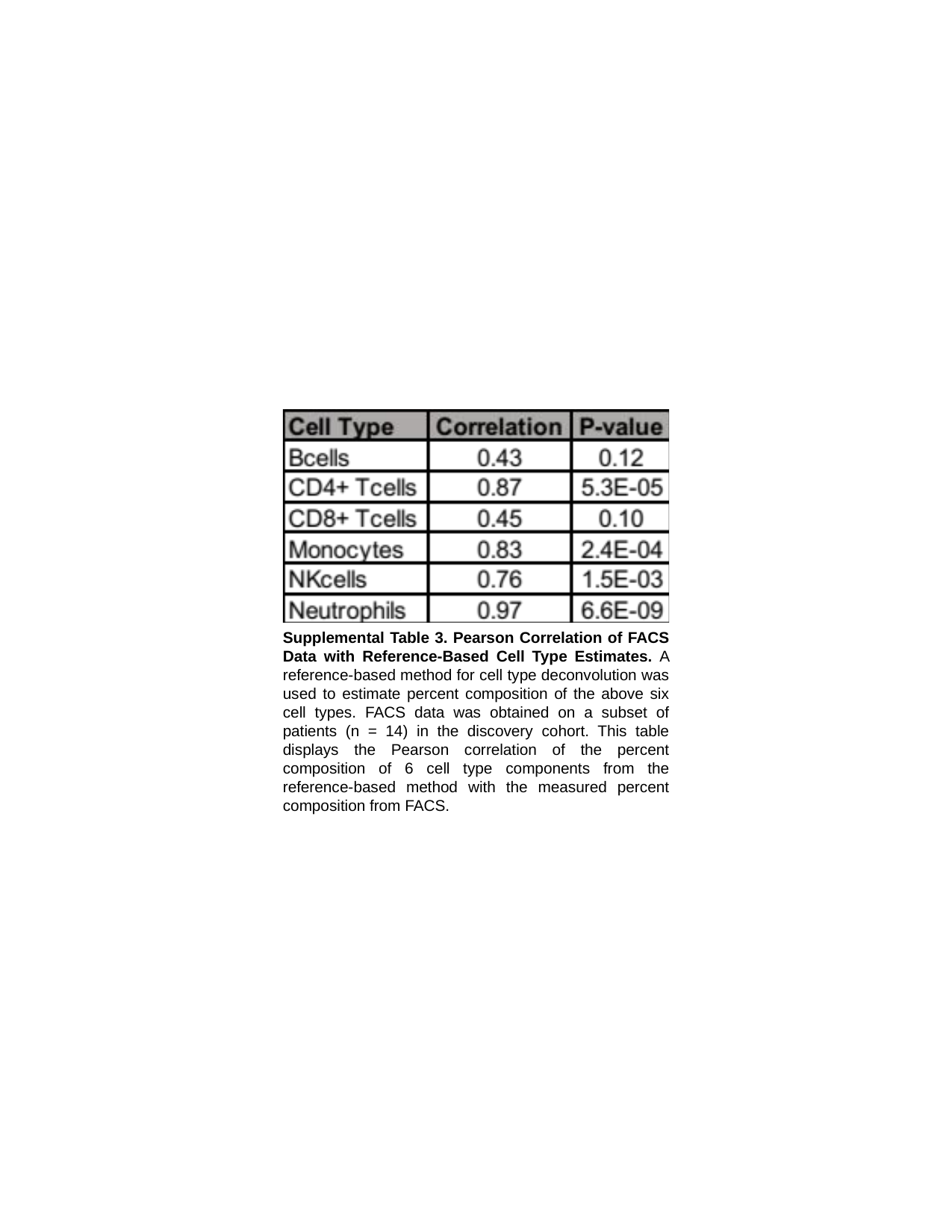

Supplemental Table 3. Pearson Correlation of FACS Data with Reference-Based Cell Type Estimates. A reference-based method for cell type deconvolution was used to estimate percent composition of the above six cell types. FACS data was obtained on a subset of patients (n = 14) in the discovery cohort. This table displays the Pearson correlation of the percent composition of 6 cell type components from the reference-based method with the measured percent composition from FACS.

## Slide 6
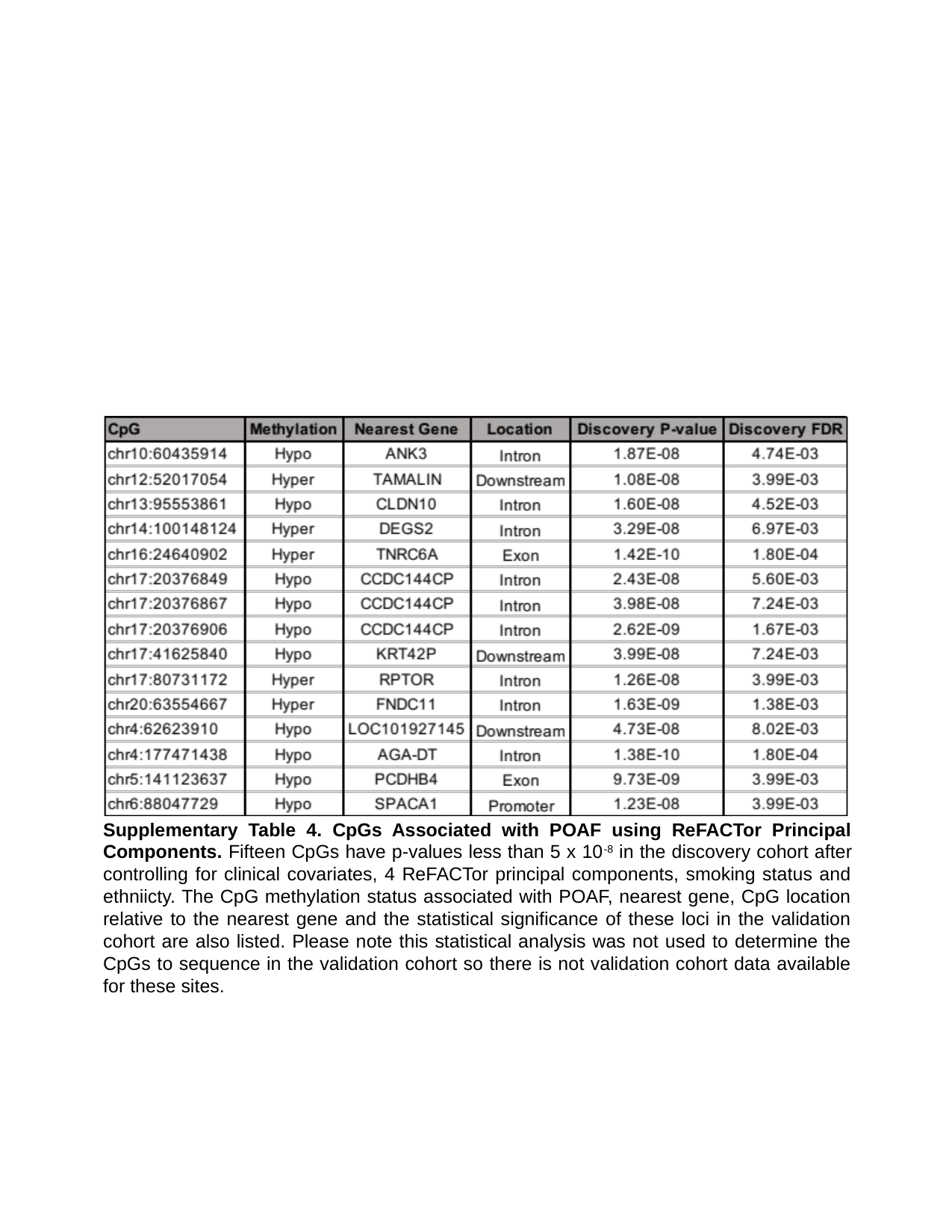

Supplementary Table 4. CpGs Associated with POAF using ReFACTor Principal Components. Fifteen CpGs have p-values less than 5 x 10^-8^ in the discovery cohort after controlling for clinical covariates, 4 ReFACTor principal components, smoking status and ethniicty. The CpG methylation status associated with POAF, nearest gene, CpG location relative to the nearest gene and the statistical significance of these loci in the validation cohort are also listed. Please note this statistical analysis was not used to determine the CpGs to sequence in the validation cohort so there is not validation cohort data available for these sites.

## Slide 7
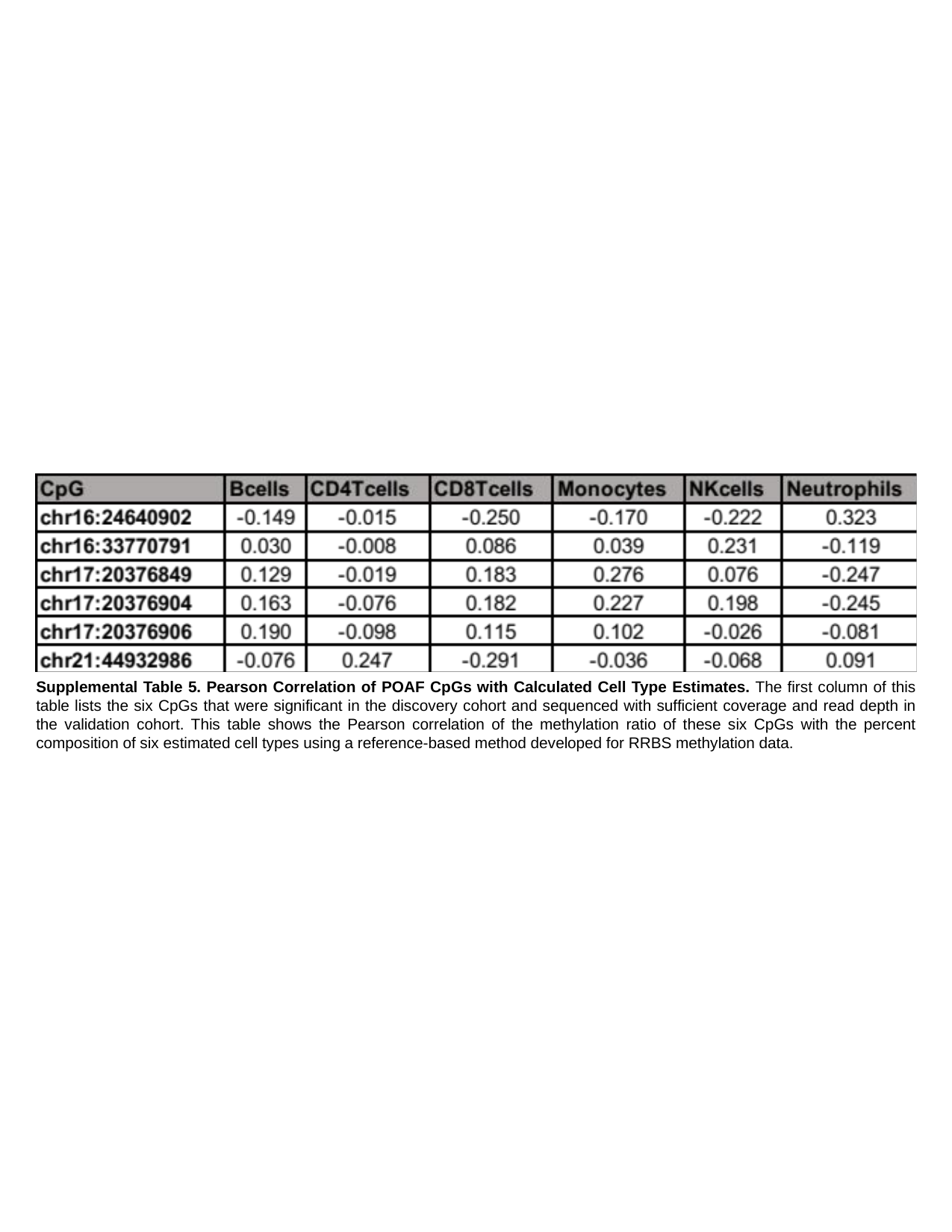

Supplemental Table 5. Pearson Correlation of POAF CpGs with Calculated Cell Type Estimates. The first column of this table lists the six CpGs that were significant in the discovery cohort and sequenced with sufficient coverage and read depth in the validation cohort. This table shows the Pearson correlation of the methylation ratio of these six CpGs with the percent composition of six estimated cell types using a reference-based method developed for RRBS methylation data.

## Slide 8
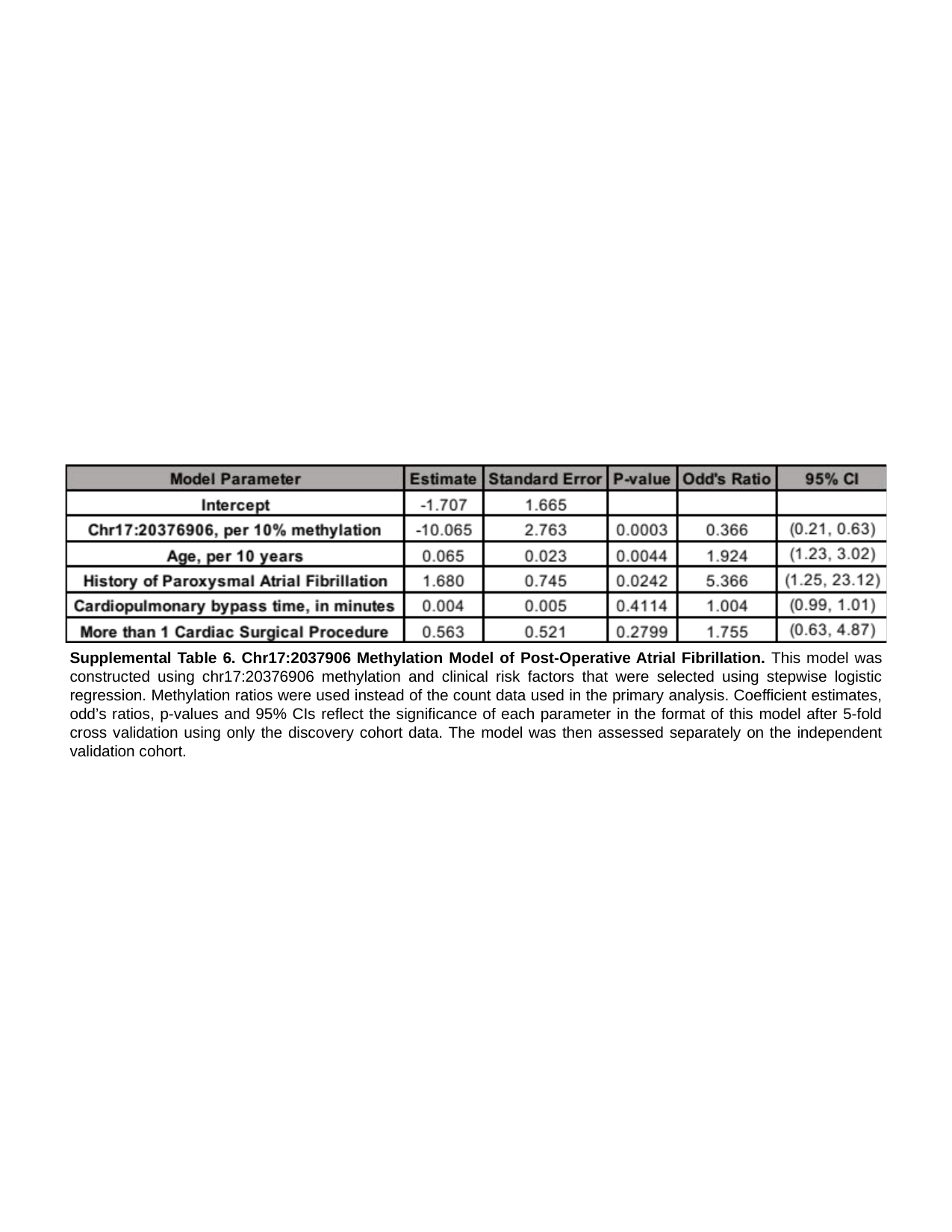

Supplemental Table 6. Chr17:2037906 Methylation Model of Post-Operative Atrial Fibrillation. This model was constructed using chr17:20376906 methylation and clinical risk factors that were selected using stepwise logistic regression. Methylation ratios were used instead of the count data used in the primary analysis. Coefficient estimates, odd’s ratios, p-values and 95% CIs reflect the significance of each parameter in the format of this model after 5-fold cross validation using only the discovery cohort data. The model was then assessed separately on the independent validation cohort.

## Slide 9
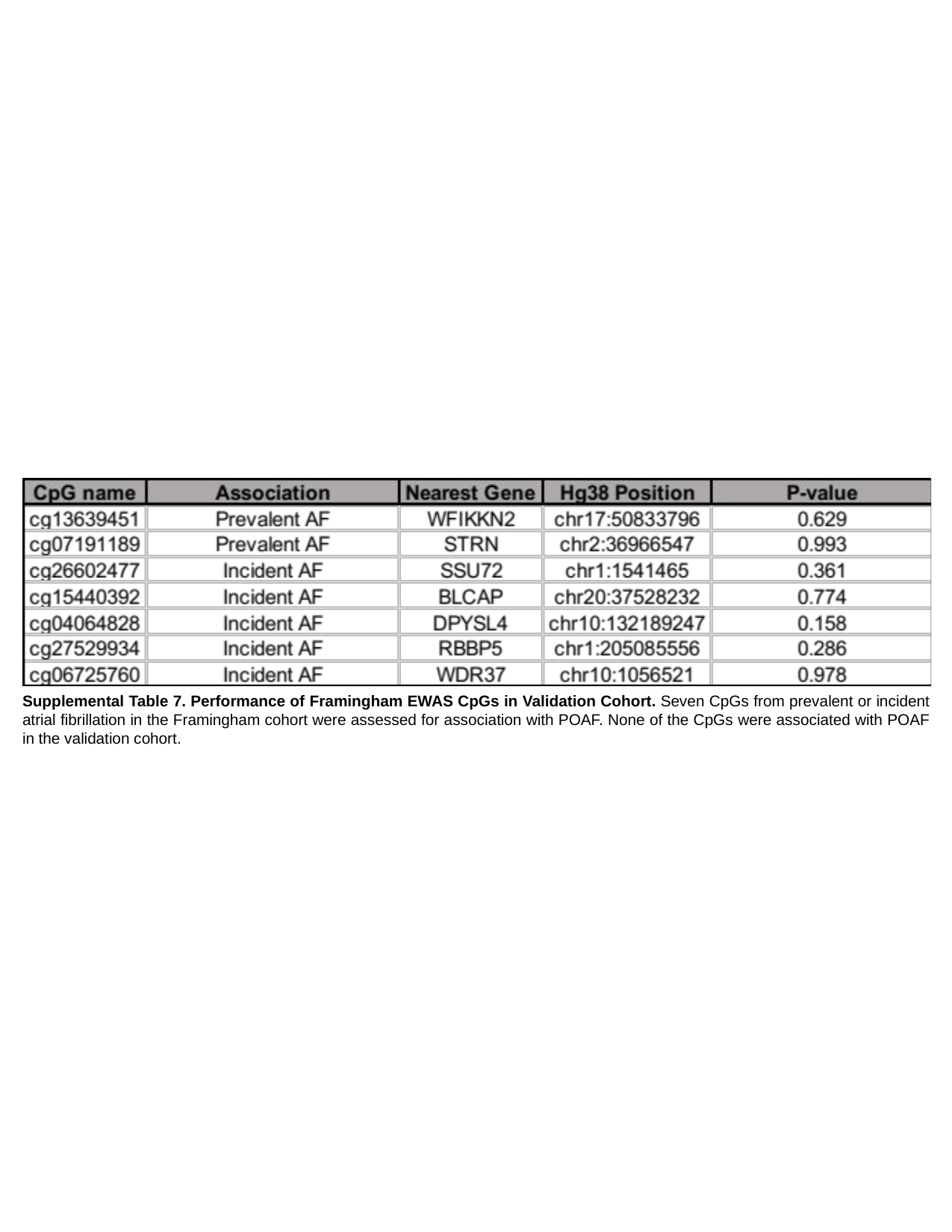

Supplemental Table 7. Performance of Framingham EWAS CpGs in Validation Cohort. Seven CpGs from prevalent or incident atrial fibrillation in the Framingham cohort were assessed for association with POAF. None of the CpGs were associated with POAF in the validation cohort.

## Slide 10
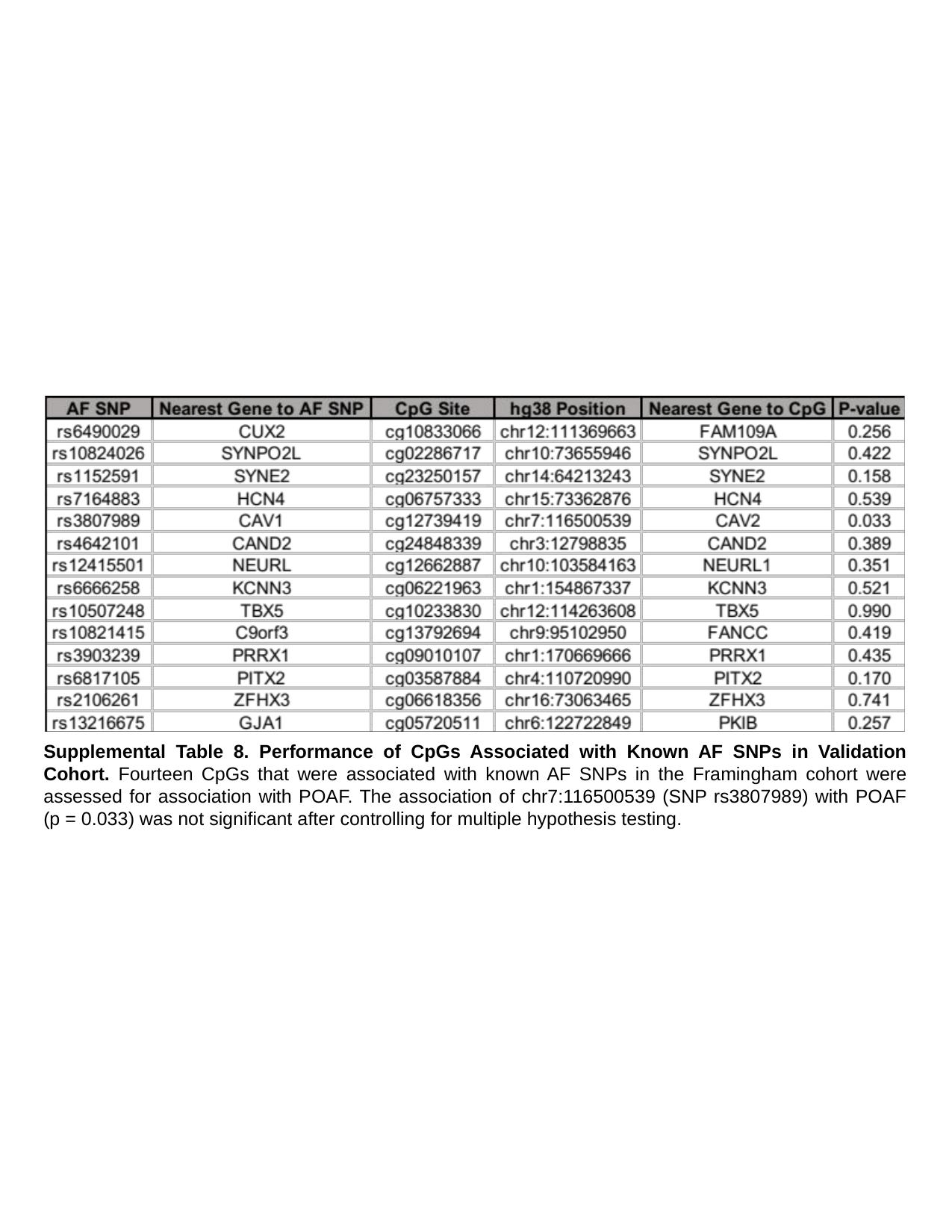

Supplemental Table 8. Performance of CpGs Associated with Known AF SNPs in Validation Cohort. Fourteen CpGs that were associated with known AF SNPs in the Framingham cohort were assessed for association with POAF. The association of chr7:116500539 (SNP rs3807989) with POAF (p = 0.033) was not significant after controlling for multiple hypothesis testing.
